# Shared book reading as a context for language intervention for children with Down syndrome: a mini-review

**DOI:** 10.3389/fpsyg.2023.1176218

**Published:** 2023-05-05

**Authors:** Mirjana Jeremic, Vesna Stojanovik, Kelly Burgoyne, Emma Pagnamenta

**Affiliations:** ^1^School of Psychology and Clinical Language Sciences, University of Reading, Reading, United Kingdom; ^2^Manchester Institute of Education, University of Manchester, Manchester, United Kingdom

**Keywords:** Down syndrome, intervention, language, communication, shared book reading

## Abstract

Acquiring language and communication skills is one of the biggest challenges for children with Down syndrome (DS). However, few evidence-based interventions exist to enhance the development of language and communication in this population. Shared book reading (SBR) is well-established as an effective intervention for language and communication development of typically developing children, and evidence of the possible effectiveness of this approach for those at risk of language difficulties is emerging. This paper provides a mini-review of the existing evidence for SBR in relation to language and communication outcomes for young children with DS. A systematic literature search was conducted with the following inclusion criteria: children with DS aged 0–6;11 years, SBR, language or communication outcomes. The results show that interventions which incorporate SBR strategies are associated with improved language and communication outcomes for young children with DS, improved parental sensitivity, and continuing implementation of SBR strategies following intervention instruction. However, evidence is limited in scope, of low quality, including mostly single case studies, with only one study having a control group. We conclude that although SBR may hold promise as a possible intervention, further research is essential to establish what specific components of SBR intervention are most effective for young children with DS and what further adaptations are needed to accommodate the cognitive profile and variability within this population.

## 1. Introduction

Down syndrome (DS) results from an extra copy of chromosome 21 and is the most common genetic cause of learning disability (Chapman and Hesketh, 2000), affecting approximately 1 in 700 live births (Martin et al., 2009). Language is often one of the biggest challenges for individuals with DS, which can sometimes be below levels expected of non-verbal mental ability (Miller, 1999). Acquiring language is often slow, with expressive vocabulary and grammar being particularly delayed (Abbeduto et al., 2007). Language ability in early childhood is a well-known predictor of later psychosocial and academic outcomes (Snowling et al., 2006), including literacy (Burgoyne et al., 2012; Hulme et al., 2012), hence providing children with DS the opportunity to advance their language skills in early development is crucial to optimize communication, educational, social and wellbeing outcomes.

Although DS is known to present with challenges with language development, few evidence-based interventions are available (O'Toole et al., 2018; Smith et al., 2020). Identifying the most effective way of involving parents/caregivers in supporting achievement of language/communication goals has been identified by the Royal College of Speech and Language Therapists as a key research priority for those with learning disabilities (RCSLT, 2019). Language interventions need to be relevant for the child’s social context and easy to implement by parents/caregivers, who are best placed to support their children’s language (Roberts et al., 2019). An intervention which is child-centered, relevant for the social context and can be delivered by parents is shared book reading.

## 1.1. Shared book reading interventions

Shared book reading (SBR) interventions build upon a natural sociocultural activity and focus on augmenting the interaction between the adult and child by using interactive book-sharing strategies, prompts and questioning (Whitehurst et al., 1988). SBR strategies include CROWD (“completion, recall, open-ended questions, wh-questions, and distancing”) questions, PEER (“prompt, evaluate, expand, repeat”) strategies (Whitehurst et al., 1994), and RAA (Read-Ask-Answer) strategies (Kent-Walsh et al., 2010). The PEER strategy has been adapted to address the needs of children with intellectual disability by adding the “extend” step (PEEER) and provide further prompts. For the purposes of this paper, we will use the term ‘SBR’ to encompass all approaches.

There is well-established evidence that SBR improves parental linguistic input, and language and pre-literacy outcomes for typically developing children and children at risk of language delay (Huebner and Payne, 2010; Aram et al., 2013; Law et al., 2018). A systematic review of 23 studies by Towson et al. (2021) examined the evidence-base for language outcomes related to SBR interventions for children with language disorder, autism, cerebral palsy, developmental delay and DS (*n* = 641, child age: 35–74 months). A range of effect sizes was reported for expressive (0.44–1.25) and receptive (0.02–1.87) language outcomes, with an overall conclusion of positive improvement and potential for SBR interventions to enhance language outcomes. Another systematic review and meta-analysis by Dowdall et al. (2020), including 19 randomized controlled trials (*n* = 2,594) targeting children aged 12–72 months with different language abilities, found that SBR interventions with more than 60 min of total intervention time yielded larger effect sizes for child language outcomes (*d* = 0.54 for expressive and *d* = 0.34 for receptive language) than those of less than 60 min (*d* = 0.41 for expressive and *d* = 0.26 for receptive language). A large effect size for caregiver competence in delivering SBR intervention was also reported (*d* = 1.01).

## 1.2. Shared book reading and Down syndrome

Whilst some studies focusing on children with developmental disabilities have included children with DS, there is to date no clear synthesis of evidence for the impact of SBR on the language skills of young children with DS. Preliminary evidence suggests that parent–child SBR interactions may be different for parents/careers and children with DS. Parents of 22 children with DS aged 22–63 months used more questions, signs, labels and grammatically simple utterances when sharing a book compared with chronologically age-matched neurotypical children. Children with DS used more nonword vocalizations and gestures, and fewer verbalizations (Barton-Hulsey et al., 2020). Similarly, children with DS have been reported to take a more passive role during reading activities when compared to their peers (van Heerden and Kritzinger, 2008; Al Otaiba et al., 2009). Given the specific behavioral profile associated with DS with a characteristic pattern of strengths and weaknesses (Fidler, 2009) there is a need to evaluate the effectiveness of SBR interventions for this particular group.

Cross-sectional studies of children with DS provide evidence for the ecological validity of SBR interventions. A study of 107 parents/careers of children with DS under the age of 7 years in the United States found that 79% had over 50 books at home and almost all read to their child daily for 10–30 min (Al Otaiba et al., 2009). Based on a survey completed by 191 parents of 1–6 year old children with DS in Ireland, Lusby and Heinz (2020) reported that most parents regularly shared books with their child, and were motivated to do so by social/emotional factors and speech and language development. Parents reported using oral language and print-referencing strategies when sharing books, but also reported challenges in engaging their child in SBR interactions and the need for guidance to enable them to support their child more effectively.

This mini review systematically synthesizes the existing evidence-base for SBR in enhancing language and communication outcomes for young children with DS aged 0–6;11 years.

## 2. Methods

A systematic literature search was conducted in January 2023 using five electronic databases (MEDLINE via PubMed, PsycINFO, Web of Science Core Collection, ERIC, Cochrane Library). The following search terms were used: [(Down syndrome OR DS OR “trisomy 21” OR disability OR Down’s syndrome) AND (“shared book read*” OR “dialogic read*” OR “interactive book read*” OR “book shar*” OR “storybook read*”)] which yielded 175 studies after removing duplicates. Titles and abstracts were independently screened for eligibility, according to the following inclusion criteria:

- Study reported results for children with DS aged between 0;0 and 6;11
- Interactive SBR included as part of the study
- Outcomes included at least one child language or communication measure (vocabulary, morphosyntax, communication)
- Published in peer-reviewed journal, in English

**Figure 1:**
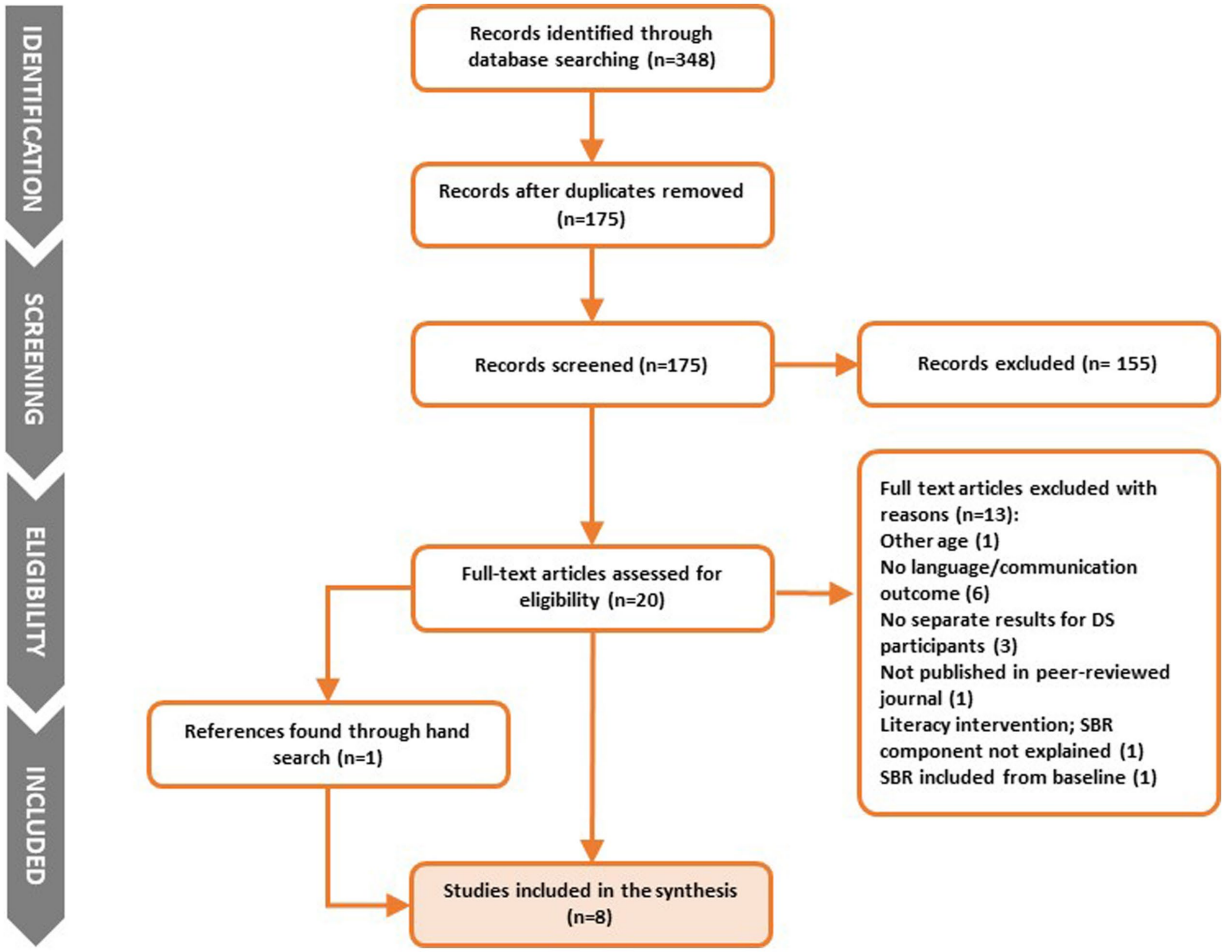
Preferred reporting items in systematic reviews and meta-analyses (PRISMA) flow diagram of the study selection procedure.

Our search identified one hundred and seventy-five studies after duplicates were removed. Of these, one hundred and fifty-five were excluded, twenty were read in full, and of these, seven met the criteria for inclusion. One study was identified through hand-searching of reference lists of the included papers (see Figure 1). From each eligible study, the following data were extracted: participant number, age and sex, study design, intervention or material modification details, study aims, parental and child outcomes, and main findings and results.

## 3. Results

Studies are summarized in Supplementary Table 1 and include five intervention studies (one SBR intervention and four combined interventions which included SBR), two experimental studies, and one observational study. Sample sizes ranged from 1 to 103 children with DS. The ages of participants ranged from 18 to 83 months.

The intervention studies included one randomized-controlled trial including a non-intervention control group (Naess et al., 2022), and four single case experimental designs (Kent-Walsh et al., 2010; Na and Wilkinson, 2019; Pierson et al., 2021; Timpe et al., 2021).

### 3.1. SBR intervention studies

Pierson et al. (2021) investigated a SBR reading intervention using a case series of four single case studies of children with developmental disabilities, including one child with DS (aged 6 years; 1 month). Caregivers received an initial one-hour training session and weekly one-hour coaching sessions (number not specified) during the intervention phase via video calls, focused on CROWD questions, PEEER strategies, and strategies to support child engagement. The parent delivered three to four reading sessions (of various length) per week to their child totaling 32 sessions. There were no significant changes in the child language outcomes as measured by correct responses to book-related questions except for an increase in the child’s comprehension of prompted questions which persisted 1 week after the intervention. There was, however, a significant increase in parental implementation of SBR strategies (see Supplementary Table 1).

### 3.2. Combined interventions including SBR

Three studies reported interventions that included SBR alongside Alternative Augmented Communication (AAC) interventions. Kent-Walsh et al. (2010) and Timpe et al. (2021) used ImPAACT (Improving Partner Applications of Augmentative Communication Techniques) in conjunction with SBR strategies. Na and Wilkinson (2019) used aided AAC modelling with a ‘Strategies for Talking about Emotions as PartnerS’ (STEPS) program within the context of book reading where parents asked questions (e.g., what, how, and why) while modelling communication about emotions. Naess et al. (2022) introduced a novel digital vocabulary intervention “Down Syndrome LanguagePlus” (DSL+) using bespoke wordless picture books with video, animation, sounds and voices. They also devised teacher manuals with scripted questions and prompts to encourage literal and inferential talk. SBR activities were combined with structured group tasks to support generalization.

The number of participants with DS ranged from one child (Kent-Walsh et al., 2010; Na and Wilkinson, 2019), three children (Timpe et al., 2021), to 103 children (Naess et al., 2022). Children were aged between 3;0 (Timpe et al., 2021) and 6;11 years (Naess et al., 2022).

Three studies involved parent-mediated interventions delivered one-to-one within the home setting (Kent-Walsh et al., 2010; Na and Wilkinson, 2019; Timpe et al., 2021). One study was classroom-based, delivered by teaching staff (Naess et al., 2022) with a combination of one-to-one, group and whole classroom sessions. Parents and teachers received training ranging from a single one-hour instructional session followed by five sessions with prompting and feedback from the clinician (Na and Wilkinson, 2019) to several hours of in-person and/or online interactive training and continuous support throughout the intervention, including the intervention materials (Timpe et al., 2021) and an intervention manual (Naess et al., 2022). The children received between 11 (Na and Wilkinson, 2019) and 75 intervention sessions (Naess et al., 2022) in total, with the story reading component often lasting about 10  minutes (Kent-Walsh et al., 2010; Timpe et al., 2021; Naess et al., 2022), and ranging between six (Na and Wilkinson, 2019) and 47  minutes (Kent-Walsh et al., 2010). The sessions were spread over a period lasting between three (Timpe et al., 2021) and 15 weeks (Naess et al., 2022). The frequency of sessions ranged from two to three times a week (Kent-Walsh et al., 2010; Na and Wilkinson, 2019; Timpe et al., 2021) to daily sessions (Naess et al., 2022).

Regarding child language and communication outcomes, Naess et al. (2022) found a significant intervention effect for trained vocabulary immediately post-intervention compared to non-intervention controls but there were no group differences on standardized vocabulary or grammar measures. Timpe et al. (2021) reported an increase in the frequency of communicative turns and novel semantic concepts recorded during reading activities post-intervention. Na and Wilkinson (2019) reported an increased number of child utterances related to the communication of emotions post-intervention, which was maintained during the generalization phase and at follow-up, 2–6 weeks later. Kent-Walsh et al. (2010) reported an increase in the total number of communicative turns and semantic concepts used post intervention which were maintained for several weeks (see Supplementary Table 1).

Parent outcomes were reported by three studies: increased accuracy in parental use of the RAA strategy post-intervention compared with baseline (Timpe et al., 2021), increase in number of open-ended questions used by the parent (Na and Wilkinson, 2019) and increase in use of communication partner interaction strategies by the parent (Kent-Walsh et al., 2010) – see Supplementary Table 1.

### 3.3. Non-intervention SBR studies

Three non-intervention studies used experimental (Burgoyne and Cain, 2022; Frizelle et al., 2022) or observational (Hilvert et al., 2022) designs to investigate SBR interactions between children with DS and their parents. The number of participants ranged from 8 to 15. Children were aged between 1;6 and 6;9 years.

Two studies adapted SBR materials to address the needs of children with DS. Burgoyne and Cain (2022) embedded 12 questions within a book to support parents to ask questions about literal and inferential information. Frizelle et al. (2022) embedded key-word signing within books to encourage child participation (signed condition) and compared it to reading a book as usual (unsigned condition). Hilvert et al. (2022) investigated the differences between maternal and paternal language input during SBR.

Differences in child language were observed in both experimental studies. Burgoyne and Cain (2022) reported that children with DS produced significantly more utterances, significantly more words and more different words when parents used question prompts compared to the typical reading condition. Frizelle et al. (2022) found that children attempted to sign significantly more in the signed than unsigned condition (see Supplementary Table 1).

Modification of materials encouraged parents to focus more on extra-textual talk (Burgoyne and Cain, 2022) and increased the number of parent utterances (Frizelle et al., 2022). Hilvert et al. (2022) found that mothers produced more utterances and used more descriptive language than fathers, while fathers read significantly more verbatim. Despite these differences, parents spent most of the book reading interaction engaged in contextualized talk (76%), followed by reading (21%), and decontextualized talk (3%) and both mothers and fathers used more complex language with children who had better language skills (see Supplementary Table 1).

## 4. Discussion

This mini-review contributes towards better understanding of the potential of SBR as a possible intervention for children with DS to enhance language and communication skills. The key findings are that interventions which incorporate SBR are associated with improved language and communication outcomes for young children with DS and that studies involving parents/careers, report changes in adult behavior and language input following the adoption of the SBR strategies. Importantly, parents/careers perceive the intervention as effective, easy to implement and enjoyable. However, the evidence is limited in scope, largely of low quality with only one intervention study including a control group. SBR is often combined with other interventions, making it difficult to identify any unique effects on language outcomes that may be attributable to SBR, but also suggesting that SBR strategies may be beneficial if used in combination with another intervention to enhance children’s language and communication skills. Non-intervention experimental and observation studies provide some support for the potential of SBR to enhance language and communication outcomes for children with DS, with evidence of question prompts and the use of key-word signing in SBR being associated with increased child participation and communication. These findings are consistent with findings of previous reviews of SBR with other populations (Mol et al., 2009; Dowdall et al., 2020; Towson et al., 2021). Parents often lack in confidence and seek advice on how to optimize these interactions with their children, and manage their child’s attention and engagement (Barton-Hulsey et al., 2020; Lusby and Heinz, 2020). This highlights the need for parent/career support for SBR, and for further research to identify effective ways to enable parents to support their child’s attention, behavior and cognitive needs during SBR activities.

Expressive language is typically an area of relative weakness in children with DS, compared with receptive language (Seager et al., 2022). This mini-review identifies increases in children’s expressive language following SBR (Na and Wilkinson, 2019; Timpe et al., 2021; Naess et al., 2022) which is also supported by existing reviews (Dowdall et al., 2020; Towson et al., 2021). This could be because SBR strategies aim to encourage children to take an active communicative role, and provide opportunities for parents to model and scaffold language in a naturally occurring context (Mol et al., 2008; Towson et al., 2021; Burgoyne and Cain, 2022). Previous studies report large effect size ranges for language outcomes which could be due to different research designs and/or measures used; this further suggests the need for future research to establish which SBR components promote improvement in language outcomes for different populations (Dowdall et al., 2020; Towson et al., 2021).

This review shows that SBR strategies have been implemented through the instruction of parents/careers/educators which can lead to behavior modification in the adult and this in turn can have an effect on the language and communication outcomes of the children with DS. This suggests effective implementation within the child’s natural environments, thus emphasizing the potential for SBR strategies to generalize beyond the intervention sessions. Involving parents/careers is essential to enable the creation of a child and family-centered intervention (Alsem et al., 2017) and SBR naturally lends itself to this approach. It should be noted that parental input may vary between mothers and fathers during SBR (Hilvert et al., 2022), and that parents adapt their language according to their child’s chronological age and language ability (Lusby and Heinz, 2020; Hilvert et al., 2022). This needs to be further explored with more controlled studies examining the possible relation between differences in parental input during SBR and child language outcomes.

Given the cognitive profile and variability that exists within the DS population (Onnivello et al., 2022), it is possible that some children may need different levels or types of adult support, specific dosage or implementation adaptations (Burgoyne and Cain, 2022). Other reviews have identified incomplete reporting of child and adult demographics including ethnicity and home language, child intellectual abilities and additional diagnosis to be the limiting factors when synthesizing effectiveness of SBR interventions (Dowdall et al., 2020; Towson et al., 2021). Burgoyne and Cain (2022) found considerable variability in parent shared reading behaviors and child engagement. They note a case of a younger child who spent less time engaging in extra-textual talk and produced less language when sharing a book with embedded prompts. This was in contrast with the behavior noted in the older children who engaged better and produced more language when parents made reading more interactive. This suggests that SBR strategies may need to be modified and adapted for children of different ages and/or attention and language skills to engage with SBR. Small-scale research has suggested that incorporating pause time (Towson et al., 2021), pictures (Whalon et al., 2013), prompts (Burgoyne and Cain, 2022) and technology enhancement (Grygas Coogle et al., 2018; Quinn et al., 2020; Naess et al., 2022) may be effective strategies in SBR with children with developmental disabilities. Moreover, interventions included in this mini-review were of variable dosage (between 11 and 75 intervention sessions in total) and dosage has been found to mediate SBR intervention effectiveness (Dowdall et al., 2020). However, due to the heterogeneity of the reported outcomes, the variability of the measures used and the fact that few studies reported actual effect sizes (see Supplementary Table 1), it is difficult to estimate for our set of studies whether dosage may have mediated the effectiveness of SBR interventions. Future research should consider the optimum dosage of intervention, which may vary among different groups. Furthermore, most studies included here measured outcomes during, or immediately after, the intervention. This lack of longer-term follow-up results means that evidence of lasting effects is currently missing and future research should bridge this gap to inform SBR practices for children with DS.

Although it is difficult to draw definitive conclusions based on the limited available evidence, the studies included in this mini-review suggest that SBR is a promising intervention approach which could be implemented with children with DS to enhance their language and communication skills.

## Author contributions

MJ, EP, VS, and KB contributed to the conception and design of the study. MJ conducted the literature searches and data extraction. EP and MJ wrote the first draft of the manuscript. All authors contributed to manuscript, read, and approved the submitted version.

## Funding

This work is supported by the Economic and Social Research Council (award no. ES/P00072X/1) in partnership with Down Syndrome Education International.

## Conflict of interest

The authors declare that the research was conducted in the absence of any commercial or financial relationships that could be construed as a potential conflict of interest.

## Publisher’s note

All claims expressed in this article are solely those of the authors and do not necessarily represent those of their affiliated organizations, or those of the publisher, the editors and the reviewers. Any product that may be evaluated in this article, or claim that may be made by its manufacturer, is not guaranteed or endorsed by the publisher.
