# Supplementary material for: Shared book reading as a context for language intervention for children with Down syndrome: a mini-review
Source: Front Psychol. 2023 May 5;14:1176218. doi: 10.3389/fpsyg.2023.1176218 (PMC10196453; doi:10.3389/fpsyg.2023.1176218)
Supplement: Supplementary file 1 [file Table_1.DOCX]

**Table 1**: Table of studies reviewed (results reported for DS participants aged between 0;0 and 6;11 and their parents)

| **Authors (year), study design** | **Ppt no, place, language** | **Child age at intake** | **Intervention or SBR modification; intervention duration, delivery mode** | **Aims** | **Measures and outcomes: parents** | **Measures and outcomes: child** | **Main findings and results** |
| --- | --- | --- | --- | --- | --- | --- | --- |
| Burgoyne and Cain  (2020)  Repeated measures experimental study | 8 children with DS (4 male and 4 female)  8 parents of children with DS (all female)  England, UK, English. | Child age: 4;7 to 6;9 | **SBR modification:** 12 question prompts embedded in the book, asking about literal and inferential information.  Parent-child dyads were videoed reading two books: one with question prompts (prompted condition) and another one in its original form (unprompted control condition).  Location:  Children’s homes | To examine the effect of prompts on the shared reading interactions of parents and young children with DS. | Parent’s language production in the context of shared book reading: total number of parent utterances during the shared reading interaction. Also, parent:child participation ratio calculated. | Children’s language production in the context of shared reading: total number of utterances, total number of words spoken, mean length of utterance (in words; MLU) and number of different words spoken. | Transcript from video was analysed using the Computerized Language Analysis Program (CLAN; MacWhinney, 1995). The parent:child participation ratio calculated using Crain-Thoreson and Dale (1999): a ratio of .50, reflecting equal participation.  As a group, just under a third of the typical shared reading session focused on **extra-textual talk** (28.26%) which increased to just under half of the session in the prompted condition (44.45%); a difference which was significant (t(7) = −2.47, p = .022, d = .87). A series of (one-tailed) paired samples t-tests showed that, as a group, children produced significantly more **utterances** (t(7) = −2.59, p = .018, d = .92) and a significantly greater **number of total words** (t(7) = −2.42, p = .023, d= .86) and greater number of different words (t(7) = −2.60, p = .018, d = .92) in the prompted condition than in the typical reading condition. Children’s **relative participation** also increased significantly in the prompted condition (t(7) = −2.34, p = .026, d = .83). There were no significant differences in **MLU** (t(7) = −0.43, p = 0.34, d = .15). |
| Frizelle et al. (2023)  Repeated measures experimental study | 36 children with DS (20 male, 16 female)  36 parents of children with DS (all female)  Ireland, Irish. | Child age: 1;6 to 5;11 | **SBR modification**:  key word signing embedded into books as a SBR technique  Parent-child dyads were videoed reading two books, one in a signed and one in an unsigned condition. | To compare two methods of a shared book reading activity - one in which a book had key-word sign prompts embedded (signed condition) and the other in which a book was read as normal (unsigned condition). | Total number of utterances; mean length of utterance (MLU) in morphemes; and vocabulary diversity. | Total number of signs produced in each condition and levels of attention and initiation as measured by the Pivotal Behaviour Rating Scale (PVBRS) (Mahoney, 1998). | Utterances transcribed using the Codes for Human Analysis Transcripts (CHAT) software. Contrasts in outcomes estimated by Poisson and linear mixed-effects models.  **Children attempted to sign significantly more in the signed than unsigned condition:** children used an average of six signs in the signed condition compared to one sign in the unsigned condition. (the incidence rate ratio (IRR) for signed vs unsigned was 5.84, p < .001), as well as showing significant increases in their levels of attention and initiation (mean difference for attention was 0.38 units, p = 0.002; mean difference for initiation was 0.35 units, p = 0.007).  **For parents, there was a significant increase in the total number of utterances used in the signed vs. unsigned condition** (IRR 1.26, p < 0.001) and a decrease in MLU (a difference in means of -0.43 units, p = 0.012). Vocabulary diversity was similar in both conditions.  Results showed that the signed condition was associated with a 26% **increase in the number of parental utterances used** (IRR 1.26, 95% CI 1.18  to 1.34, p < 0.001). This increase was evident in 26  of the 36 parent participants. The signed condition  was also associated with a **reduction in mean MLU in morphemes** (a difference in means of −0.43 units, 95% CI −0.74 to −0.11, p = 0.012), evident for 22 of the 36 participants. Mean vocabulary diversity was lower in the signed condition, but the difference was non-significant (a difference in means of −2.13 units, 95% CI −5.19 to 0.93, p = 0.177). |
| Hilvert et al. (2022)  Group comparison observational study | 15 children with DS (8 males and 7 females)  15 mothers and fathers of children with DS  Madison, Wisconsin, USA.  English. | Child age: 2;0-5;1 | 8 min language samples obtained from video recordings of mother-child and father-child dyads separately engaging in book reading. Mothers and fathers were provided with the same five board books. | To evaluate the quantity and quality of language input among mothers and fathers of young children with DS during book reading interactions and investigate associations with child language. | Total number of analysed utterances, grammatical complexity (i.e., mean length of utterance in morphemes [MLU]), lexical diversity (i.e., number of different words [NDW]), as well as the percent of intelligible utterances. | Total number of analysed utterances, grammatical complexity (i.e., mean length of utterance in morphemes [MLU]), lexical diversity (i.e., number of different words [NDW]), as well as the percent of intelligible utterances. | **Differences between mother and father input**  Paired samples t-tests revealed that mothers produced more utterances (t(14) = - 2.26, p = 0. .042) and used a higher proportion of descriptive language (t(14) = - 2.21, p = 0. .046) than fathers. However, fathers used a higher proportion of reading utterances (t(14) = 2.38, p = 0. .033). Parents did not differ on any other type of input.  Despite these differences, parents spent most of the book reading interaction engaged in contextualized talk (76%), followed by reading (21%), and decontextualized talk (3%).  **Correlations between child chronological age and parent input**  A positive association was found between child chronological age and the proportion of paternal decontextualized input.  **Correlations between child language and parent input**  Both fathers and mothers used more complex language with children who had better language skills.  **Child language during book reading**  Child grammatical complexity and lexical diversity were positively related to maternal lexical diversity. Similarly, child grammatical complexity was positively related to the proportion of maternal reading. Moreover, child lexical diversity was positively related to paternal decontextualized input. |
| Kent-Walsh et al. (2010)  Multiple-probe-across-participants design | 3 children with DS (male)  3 children with non-DS diagnoses excluded from the results analysis  3 parents of children with DS (female)  3 parents of children with non-DS diagnosis were excluded from the analysis  Central Florida, Orlando,  English. | DS child age: 4;7-5;11  Non-DS child age:  5;0-8;3 | **Intervention:**  ImPAACT (Improving  Partner Applications of Augmentative Communication  Techniques) Program with storybooks as a  primary context for communication. RAA technique + providing an aided AAC model with expectant delays.  **Intervention duration:**  Parent component: 2-2.5hrs over 1-2 week period.  Child component: a mean of 27 min (range = 17–47 min) of story book reading during parent training plus subsequent 13 10-min storybook-reading sessions including baseline, intervention, generalisation phase which was 1-2 weeks after intervention and maintenance phase which was 2, 4 and 8 weeks after generalisation).  **Intervention delivery:**  parent-mediated in participant home. | To investigate the effects of a communication partner instruction strategy for parents of children using augmentative and  alternative communication (AAC) on the communicative  turn taking of their children within the storybook-reading contexts. | Application of the communication partner interaction strategy. | Total number of communicative turns (speech, aided AAC, manual signs, head nods/shakes, and other gestures  such as pointing to pictures in the story) during 10min reading session,  and the expression of different semantic concepts  in children using AAC. | **Parent results:** none of the mothers used communication partner interaction strategy during baseline sessions (0%) but all the mothers (including mothers of children with DS) used the strategy with at least 90% accuracy during the intervention, generalisation (1-2 weeks after intervention) and maintenance (2, 4 and 8 weeks after generalisation) phase.  **Child results**: The number of turns that each child took during the first intervention session more than doubled, compared with each child’s highest number of turns for any baseline session. The ratios of the highest number of turns during a baseline session compared with the number of turns during the first intervention session for three children with DS ranged from 1 to 6; 1 to 14; and 1 to 16. The majority of their turns involved the expression of different concepts for each session. All children (including children with DS) demonstrated improvement rate differences between the baseline phases and each remaining phase (intervention, generalisation, maintenance) at least 80% indicating that the intervention had a large effect for all children. Children required only minimal prompting before taking turns. |
| Naess et al. (2022)  Randomised controlled trial | 103 children with DS: 50 in the intervention group (28 males, 22 females) and 53 in control (26 males, 27 females).  Norway, Norwegian. | Intervention group mean age in months = 76.84, SD =5.20  Control group mean  age in months = 77.45, SD = 5.60 | **Intervention:** Down Syndrome LanguagePlus (DSL+, a novel digital vocabulary intervention): Bespoke wordless picture books for SBR to introduce new vocabulary delivered via iPad app; teaching materials, written manual and scripts for book dialogues provided PLUS  Structured group task to support generalisation.  **Intervention duration:**  Teacher component:  6 hours of online interactive training.  Child component:  daily sessions of approximately 15 minutes over 15 weeks (75 sessions)  **Intervention delivery:**  Teacher component:  initial training by researcher plus support throughout the intervention via telephone and email.  Child component:  mixture one-to-one (3 days a week), small-group (1 day a week), and full-class (1 day a week). | Research questions (RQ)  (RQ1): Is the intervention more effective than business as usual on measures of trained expressive vocabulary, receptive vocabulary, and vocabulary breadth and depth?  (RQ2): Is the intervention more effective than business as usual on standardized measures of expressive vocabulary, receptive vocabulary, receptive grammar, listening comprehension, and narrative skills?  (RQ3): Is there an interaction effect between posttest performance and the child factors: (a) nonverbal mental ability, (b) attention, and (c) pretest score? | N/A | Increase in receptive and expressive vocabulary breadth and depth of targeted vocabulary (bespoke assessment). Standardised vocabulary measures (British Picture Vocabulary Scale-II (BPVS) - Norwegian ver. by  Lyster et al., 2010). Standardised grammar measures (Test for Reception  of Grammar - Bishop, 2003  Norwegian ver. by  Lyster & Horn, 2009). Non-verbal ability measures (WPPSI-III Wechsler, 2002). Attention measures (Attention Behaviour Roid & Miller, 1997). | Children in the intervention group made greater gains than children in the control group (business as usual) in **expressive vocabulary breadth** (d = .429, CI [.160, .699]) and **receptive vocabulary breadth** (d = .447, CI [.193, .700]) for trained vocabulary. For **vocabulary depth** measure (how well the words are known), the results were nonsignificant, with an effect size of d = .209 (95% CI [−.108, .526]).  No generalisation effects to **expressive vocabulary** (d = −.061, 95% CI [−.246, .125]), **receptive vocabulary** (d = .154, 95% CI [−.104, .411]) or **grammar** (d = −.089, 95% CI [−.368, .190]) as measured by standardised tests.  No interaction effects on trained vocabulary were found between the treatment and the covariates of attention and nonverbal mental ability. |
| Na and Wilkinson (2019)  Multiple-probe-across-participants design | 1 child with DS (male)  2 other children with DS were older than 6;11 therefore excluded from the results analysis  1 parent (female)  Pennsylvania, United States. | Child age: 5;1  Excluded children age: 8;9 and 9;8 | **Intervention:**  Parent trained to design an emotion communication board to use for aided AAC modelling, and emotions communicating by child. PLUS  Strategies for Talking about Emotions as PartnerS (STEPS) programme within the context of book reading (parents ask questions (e.g. what, how, and why) while providing and modelling labels for emotions).  **Intervention duration:**  Parent component: 1-hour instructional session.  Child component: 5 parent-mediated post-intervention sessions with prompting and feedback from the clinician. This was followed by generalisation phase (one week after post-intervention phase) and maintenance phase (2–6 weeks following completion of  the generalisation phase with 1–2 weeks apart). Reading sessions (three videorecorded probes for each phase) lasted on average 6min:26sec and were of comparable length.  **Intervention delivery:**  parent-mediated in participant home. | To investigate the effect of STEPS programme on parents' provision of opportunities for emotion communication using visual communication supports. | Provision of open-ended questions on each of the three steps of emotion communication (label,  reason, solution) with the use of emotion boards during storybook reading. | Frequency  of emotion-related utterances. | Each entire reading session was used as a unit of analysis for coding. Inspection of the data graphed for visual analysis shows a clear and immediate shift between baseline and post-intervention phases.  **For parent measures**, zero was recorded at baseline for number of open-ended questions for all three steps (label, reason, solution) and between 1 and 5 in the first post-instruction session. This effect was maintained during the generalisation phase (2-4) which had a modified book material and maintenance phase (2-5) which was identical to baseline.  **Data for the child** number of utterances related to the emotion communication increased from baseline (0-2) to post-intervention (2-9), showing immediacy of the effect. This effect was maintained during the generalisation phase one week post intervention (2-7) and maintenance phase two to six weeks after generalisation (2-11). |
| Pierson et al. (2021)  Multiple-probe-across-participants design | 1 child with DS (male)  3 children with non-DS diagnosis were excluded from the analysis  1 parent (female)  3 parents of children with non-DS diagnosis were excluded from the analysis  South central United States, English. | Child with DS age: 6;1  Non-DS diagnosis child age:  5;0-7;3 | **Intervention:** PEEER strategy and CROWD questions including video feedback from the researcher.  **Intervention duration:**  Parent component: one 60min parent training intervention session via telepractice plus 60min weekly coaching sessions.  Child component: parent delivered three to four book reading sessions (of various length) per week to their child totalling 32 sessions.  **Intervention delivery:**  Parent component: via telepractice.  Child component:  parent-mediated in child’s home. | To investigate  the effect of parent coaching on the parent implementation of modified shared book reading procedures and child language skills; to investigate social validity of the intervention (parent opinions of the goals, outcomes, and intervention procedures). | Number of PEEER strategies the parent implemented relative to the number of opportunities for the strategies implementation; the number of modified dialogic reading component implementation opportunities missed per condition, and the percentage of components correctly implemented. | Number of comprehension questions the child answered correctly (correct, prompted  correct, prompted incorrect, or incorrect) during the book reading activity with the parent. | **Results for parent implementation:**  A functional relation was observed between parent implementation and coaching. For parent PEEER strategies, implementation 0.96 effect was observed indicating a strong to very strong effect on parent implementation of modified SBR procedures. For modified SBR components opportunities parent implemented 41% correctly in baseline, 80% were implemented correctly in intervention which dropped to 55% and %74 during the maintenance phase (one week after the intervention).  **Results (percentage) for child correct responses to comprehension questions:**  In baseline, data have no variability or trend for the unprompted responses to the comprehension questions (average = 2%, range: 0%-10%). An immediacy of effect is not present between baseline and intervention. Intervention data have no trend or variability (average = 2%, range: 0%-10%). Generalisation data (collected using family books without prompt questions) are 0% in baseline, while data in intervention are 10%, 10%, and 50%.  Data for prompted correct responses to comprehension questions in baseline are variable and have no trend (average = 9%, range: 0%-20%). An immediacy of effect was noted between baseline and intervention conditions. Intervention data are stable for the first three data points and can be characterized by a descending trend for the last three data points (average = 96%, range: 80%-100%). Generalisation data are 80% in baseline, while data in intervention are 90%, 90%, and 40%. In the maintenance condition (collected one week after the intervention), correct data were 0% and 50% (generalisation), while prompted correct data were 100% and 40% (generalisation).  **Results for social validity of the intervention:**  parent reports that intervention was beneficial and enjoyable for the child, easy to implement, and that they were able to teach the child new important skills which they intend to contunue using (5-point Likert scale was adapted from a questionnaire developed by Roberts and Leko (2013)). |
| Timpe, E. M., et al. (2021)  Multiple-probe-across-participants design | 3 children with DS (2 male and 1 female)  3 parents (all female)  Central Florida,  English. | Child age:  3;0 to 5;11 | **Intervention:**  ImPAACT (Improving Partner Applications of Augmentative Communication Techniques; Kent-Walsh, Binger, & Malani, 2010) teach communication  partners to facilitate the early language and communication  skills of children who use AAC PLUS RAA (Read-Ask-Answer; Kent-Walsh, Binger, & Hasham, 2010)  **Intervention duration:**  Parent component: nine parent training sessions over three consecutive weeks (six in-person at 60 min each and three telepractice at 30 min each)  Child component: Three weeks of reading sessions lasting minimum of 10min (including baseline, intervention, post-intervention, generalisation, and maintenance sessions).  **Delivery:**  Parent component: researcher delivered training; Child component: parent-delivered  Hybrid model of telepractice and in person at university speech and hearing clinic and participants’ homes. | To assess parents’ accurate implementation of the instructional strategy and children’s communicative turns. | Parent acquisition of the instructional strategy—specifically, percentage of accurate use of the strategy. | Child participant’s communication measured as turn-taking rates; vocabulary diversity measured as frequency of novel semantic concepts. | Measures coded from videorecorded 9min middle segment of 10min parent-child interactions  Improvement rate differences (IRD) were calculated as an effect size indicator; scores greater than 0.75 were considered very large effect sizes.  **Parents’ implementation of the Read–Ask–Answer strategy**  During baseline, the parents seldom used the RAA strategy steps accurately (range: 0–8%). In the post-intervention measures all parents achieved accuracy levels at or above 80%; scores were 1.0 for baseline versus post-intervention phases for all of the parent data, that is, very large effects.  Generalised use of the RAA strategy were recorded at high levels of accuracy of the strategy use (range: 63–100% accurate implementation). IRD scores were 1.0 for baseline versus generalisation phases for all of the parent data (very large effects).  At the maintenance stage, the use of the instructional strategy was in 100% of opportunities, IRD scores were 1.0 for baseline versus maintenance phases for all parent data (very large effects).  **Children’s turn-taking**  During baseline, recorded at 0 to 7 per 9-min session. Post-intervention rates ranged from 10 to 110 turns per 9-min session. IRD scores were 1.0 for baseline versus post-intervention phases for all of the child data, (a very large intervention effect)  During the baseline phase for the generalisation books, all of the child participants maintained low turn-taking rates (range: 0–12 turns/session), with marked increases during the post-intervention and generalisation phase (range: 5–116 turns/session). IRD scores were1.0 for baseline versus generalisation phases for all of the child data, indicating a very large intervention effect.  Compared with baseline levels, the level of the data increased during maintenance in all cases (28–63 turns per session for the dependent variable book series and 30–80 turns for the generalisation series). IRD scores from baseline to maintenance were 1.0 for both conditions.  **Frequency of novel semantic concepts**  The overall number of concepts ranged from 0 to six in baseline vs. six to 47 in post-intervention and nine to 33 in maintenance. For the generalisation measures, the number of novel concepts ranged from 0 to nine in baseline, six to 44 in the generalisation phase, and 10 to 50 in the maintenance phase. IRD scores from baseline versus post-intervention, generalisation, and maintenance were 1.0 in all cases. |
